# Elucidation of ubiquitin-conjugating enzymes that interact with RBR-type ubiquitin ligases using a liquid–liquid phase separation–based method

**DOI:** 10.1016/j.jbc.2022.102822

**Published:** 2022-12-21

**Authors:** Ryota Hayashida, Reika Kikuchi, Kenichiro Imai, Waka Kojima, Tatsuya Yamada, Miho Iijima, Hiromi Sesaki, Keiji Tanaka, Noriyuki Matsuda, Koji Yamano

**Affiliations:** 1 Ubiquitin Project, Tokyo Metropolitan Institute of Medical Science, Tokyo, Japan; 2 Department of Computational Biology and Medical Sciences, Graduate School of Frontier Sciences, The University of Tokyo, Kashiwa, Chiba, Japan; 3 Department of Biomolecular Pathogenesis, Medical Research Institute, Tokyo Medical and Dental University, Tokyo, Japan; 4 Cellular and Molecular Biotechnology Research Institute, National Institute of Advanced Industrial Science and Technology (AIST), Tokyo, Japan; 5 Department of Cell Biology, Johns Hopkins University School of Medicine, Baltimore, Maryland, USA; 6 Protein Metabolism Project, Tokyo Metropolitan Institute of Medical Science, Tokyo, Japan

**Keywords:** mitochondria, mitophagy, Parkin, PINK1, RBR-type E3, ubiquitin, Ash, Assembly helper, ER, endoplasmic reticulum, Fluoppi, fluorescence-based technology detecting PPI, GST, glutathione-*S*-transferase, HA, hemagglutinin, hAG, homotetramer forming Azami-Green, JSPS, Japan Society for the Promotion of Science, LLPS, liquid–liquid phase separation, LUBAC, linear Ub chain assembly complex, OMM, outer mitochondrial membrane, PPI, protein–protein interaction, RBR, RING-between RING, TBS, Tris-buffered saline, TCEP, Tris(2-carboxyethyl)phosphine, TNF-α, tumor necrosis factor alpha, Ub, ubiquitin

## Abstract

RING-between RING (RBR)-type ubiquitin (Ub) ligases (E3s) such as Parkin receive Ub from Ub-conjugating enzymes (E2s) in response to ligase activation. However, the specific E2s that transfer Ub to each RBR-type ligase are largely unknown because of insufficient methods for monitoring their interaction. To address this problem, we have developed a method that detects intracellular interactions between E2s and activated Parkin. Fluorescent homotetramer Azami-Green fused with E2 and oligomeric Ash (Assembly helper) fused with Parkin form a liquid–liquid phase separation (LLPS) in cells only when E2 and Parkin interact. Using this method, we identified multiple E2s interacting with activated Parkin on damaged mitochondria during mitophagy. Combined with *in vitro* ubiquitination assays and bioinformatics, these findings revealed an underlying consensus sequence for E2 interactions with activated Parkin. Application of this method to other RBR-type E3s including HOIP, HHARI, and TRIAD1 revealed that HOIP forms an LLPS with its substrate NEMO in response to a proinflammatory cytokine and that HHARI and TRIAD1 form a cytosolic LLPS independent of Ub-like protein NEDD8. Since an E2–E3 interaction is a prerequisite for RBR-type E3 activation and subsequent substrate ubiquitination, the method we have established here can be an in-cell tool to elucidate the potentially novel mechanisms involved in RBR-type E3s.

Ubiquitination plays critical role in not only protein degradation *via* proteasomes but also cell cycle progression, immune responses, and organellar degradation *via* autophagy. Ubiquitin (Ub) is a small 76-amino acid protein that post-translationally modifies substrate proteins *via* conjugation of the Ub C-terminal Gly residue to a substrate Lys residue. In addition, Ub can generate unique Ub linkages by similarly conjugating to other Ub molecules through any of its seven Lys residues or N-terminal Met. Conjugation occurs in a sequential reaction mediated by Ub-activating (E1), Ub-conjugating (E2), and Ub ligase (E3) enzymes. The initiating E1 conjugation to the Ub C-terminal Gly is ATP dependent. Ub is then transferred to a conserved catalytic Cys residue in E2 *via* a transthiolation reaction to generate E2∼Ub. Finally, E3 facilitates the transfer of Ub from E2 to the substrate.

Although only two Ub-specific E1 enzymes, UBA1 and UBA6, have been identified, ∼30 E2 and ∼600 E3 enzymes are encoded in the human genome. E3 enzymes have been classified into four families: RING-type, HECT-type, U-box-type, and RING-between RING (RBR)-type. While RING-type E3s function as a scaffold allowing the direct transfer of Ub from E2 to substrates, HECT E3s contain a catalytic Cys residue in their HECT domain that receives Ub *via* a thioester-linked and transfer it to their substrate. RBR-type E3s, which possess two RING-like domains (RING1 and RING2), were initially thought to be a subtype of RING E3s. However, Wenzel *et al.* (1) found that although RING1 interacts with E2∼Ub, RING2 has a Cys residue that receives Ub from an E2, similar to the HECT domain. Structural and biochemical analyses, which were later published, support this finding (2, 3, 4). Another interesting feature of RBR-type E3 is that the ligase activity is regulated by intra- and inter-protein–protein interactions (PPIs). Indeed, most of the RBR-type E3s are multidomain proteins with various interactions (5, 6).

Parkin, the gene product mutated in familial Parkinson’s disease, is one of the most characterized RBR-type E3 ligases (7, 8). Cytosolic Parkin is recruited onto dysfunctional mitochondria in response to losses in the inner mitochondrial membrane potential (9). This mitochondrial localization is facilitated by PINK1, another gene product mutated in recessive forms of familial Parkinson’s disease that accumulates only on the outer membrane of dysfunctional mitochondria (10, 11, 12) and phosphorylates Ub (13, 14, 15). The mitochondrial-localized phosphorylated Ub strongly binds Parkin, thus tethering cytosolic Parkin to the dysfunctional mitochondria (16, 17, 18, 19). PINK1 also phosphorylates a Ub-like domain in Parkin (20, 21), which leads to the exposure of an E2 binding site in the RING1 domain and a catalytic Cys residue (C431) in the RING2 domain, thereby releasing the E3 enzyme from its autoinhibition state and making it enzymatically active (22, 23). Activated Parkin then ubiquitinates various outer mitochondrial membrane (OMM) proteins such as TOMM20 and VDAC. Importantly, the activities catalyzed by Parkin, PINK1, and phosphorylated Ub form a positive feedback loop that amplifies both Parkin recruitment and poly-Ub chain formation on the mitochondria (16, 17).

Several studies have focused on elucidating the E2 enzymes involved in Parkin-mediated ubiquitination. One such enzyme is UBE2L3 (also known as UbcH7), which preferentially transfers Ub to the Parkin catalytic Cys residue (1). In addition to UBE2L3, Geisler *et al.* (24) used an siRNA-based screen to show that UBE2N and UBE2D2/3 are required for mitochondrial polyubiquitination and subsequent p62 recruitment. Fiesel *et al.* (25) reported that the combined knockdown of UBE2L3, UBE2D2/3, and UBE2N blocks Parkin recruitment and degradation of mitochondrial proteins. However, Shiba-Fukushima *et al.* (26) showed that siRNA knockdown of UBE2N does not block Parkin translocation, although K63-linked Ub chains are significantly reduced. In sharp contrast, Lazarou *et al.* (27) used a cell-free ubiquitination assay to show that various E2 enzymes are involved in Parkin-dependent Ub-chain formation on damaged mitochondria. Ordureau *et al.* (16) showed *via in vitro* Parkin autoubiquitination assays that UBE2D2, UBE2E1, UBE2J2, and UBE2L3, but not UBE2N/UBE2V1 or UBE2S, induce poly-Ub chain formation on Parkin. Thus, despite intensive research, the molecular basis of E2 specificity for Parkin activation remains enigmatic. This determination is further complicated by varied expression levels of the assorted E2s in different cell types and potential compensation by redundant E2 functionalities. In addition, siRNA-based studies may be limited by potential off-target effects (exemplified by the genome-wide siRNA analyses of PINK1 (28)). Therefore, the direct detection of E2 and activated RBR-type E3 interactions in living cells was essential to study the function of E2–E3 interaction. Here, we established an in-cell–based method to capture these direct interactions.

## Results

### The E2 enzyme UBE2L3 is not stably associated on mitochondria during Parkin-driven mitophagy

**Figure 1:**
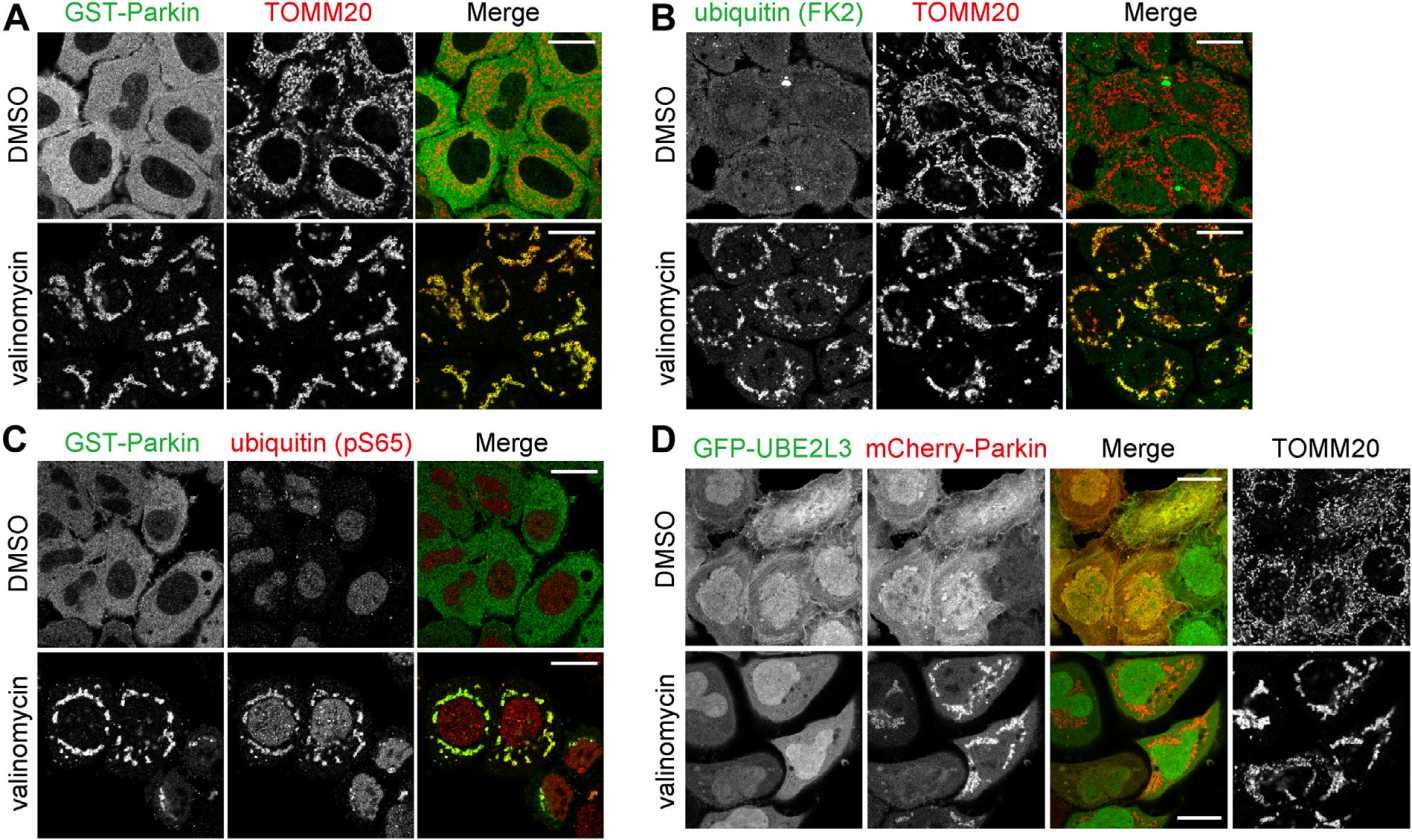
**UBE2L3 is not stably associated on mitochondria during Parkin-driven mitophagy.** HeLa cells stably expressing GST-Parkin (*A*–*C*) or HeLa cells expressing GFP-UBE2L3 and mCherry-Parkin (*D*) were treated with dimethyl sulfoxide (DMSO) or valinomycin for 3 h and immunostained with anti-GST, TOMM20, ubiquitin (FK2), and ubiquitin (pS65) antibodies. Bars represent 10 μm. GST, glutathione-*S*-transferase.

Once activated on damaged mitochondria, Parkin conjugates Ub from an E2 enzyme to Lys residues of various OMM proteins on the damaged mitochondria. Valinomycin treatment for 3 h, which causes a loss in the mitochondrial membrane potential, induced robust Parkin translocation in HeLa cells (Fig. 1*A*). Endogenous Ub, which diffusely localizes in the cytosol under basal conditions, also associated on the damaged mitochondria (Fig. 1*B*). In addition, we confirmed that phosphorylated Ub generated by PINK1 also accumulates on the damaged mitochondria (Fig. 1*C*). In sharp contrast, GFP-tagged UBE2L3, one of the E2 enzymes that interacts with activated Parkin (1), was not detected on the mitochondria (Fig. 1*D*). These results suggest that the Parkin–E2 interaction is transient, and translocation of E2 enzymes to damaged mitochondria during mitophagy cannot be captured by commonly used microscopic analysis.

### Oligomerized Parkin C431A can translocate to mitochondria without the positive-feedback amplification loop

**Figure 2:**
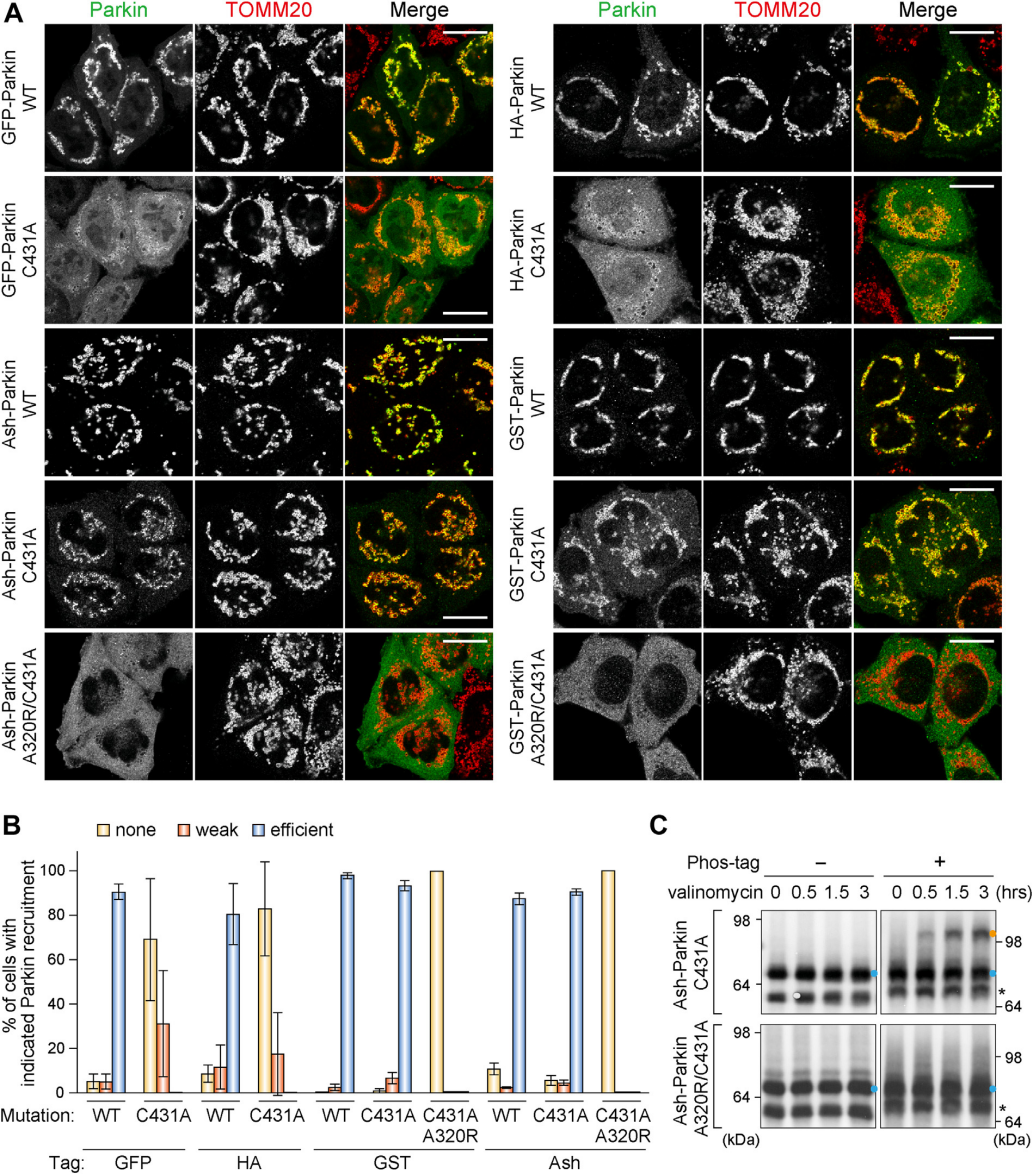
**Oligomerized Parkin can translocate to mitochondria in the absence of the ubiquitin amplification positive-feedback loop.***A*, HeLa cells expressing the indicated Parkin were treated with valinomycin for 3 h and then immunostained. Bars represent 10 μm. *B*, the percentage of cells in (*A*) exhibiting mitochondria with translocated Parkin. Error bars represent mean ± SD of >100 cells counted in each of three independent experiments. *C*, HeLa cells expressing Ash-Parkin (C431A or A320R/C431A) were treated with valinomycin for the indicated times. Total cell lysates were analyzed by phos-tag PAGE and immunoblotted with an anti-Parkin antibody (51). Unphosphorylated and phosphorylated Parkin are indicated by *light blue* and *orange dots*, respectively. *Asterisks* denote truncated Parkin species. Ash, Assembly helper.

The transient Parkin–E2 interaction could be that the E2 rapidly dissociates from activated Parkin back into the cytosol after transferring Ub. To stabilize the Parkin–E2 interaction, we blocked Ub transfer from E2 enzymes to Parkin by introducing a C431A mutation in Parkin. However, neither GFP-tagged nor hemagglutinin (HA)-tagged Parkin (C431A) mutants were as efficiently recruited to mitochondria as WT Parkin (Fig. 2, *A* and *B*), probably because the E3-inactive Parkin (C431A) mutant cannot mediate the positive feedback ubiquitination cycle. Fusion of either an Ash (Assembly helper) tag or a glutathione-*S*-transferase (GST) tag, which form homo-oligomers, to Parkin (C431A) reversed the effect and allowed translocation to mitochondria (Fig. 2, *A* and *B*). The introduction of an A320R mutation to Parkin (C431A), which abrogates the interaction with phosphorylated Ub, completely inhibited Parkin translocation to mitochondria (Fig. 2, *A* and *B*), indicating that translocation of the Ash-tagged or GST-tagged Parkin (C431A) was dependent on the phosphorylated Ub generated by PINK1 on the damaged mitochondria. Furthermore, Phos-tag analysis showed that a portion of Ash-Parkin (C431A), but not Ash-Parkin (A320R/C431A), was migrated slower, indicating that, despite the enzymatic inactive Parkin, the UBL domain is phosphorylated on the damaged mitochondria (Fig. 2*C*). This suggests that an E2-binding site in the RING1 domain of Ash-Parkin (C431A) is exposed by phosphorylation of the UBL domain. Therefore, through phosphorylated Ub, a portion of cytosolic Parkin (C431A) is recruited to damaged mitochondria, and the recruitment of other Parkin (C431A) is induced by the oligomerization. These results indicate that either Ash-tag or GST-tag oligomerized Parkin can bypass the E3 ligase activity–dependent requirement for mitochondrial translocation.

### In-cell interactions between various E2 enzymes and Parkin on damaged mitochondria

**Figure 3:**
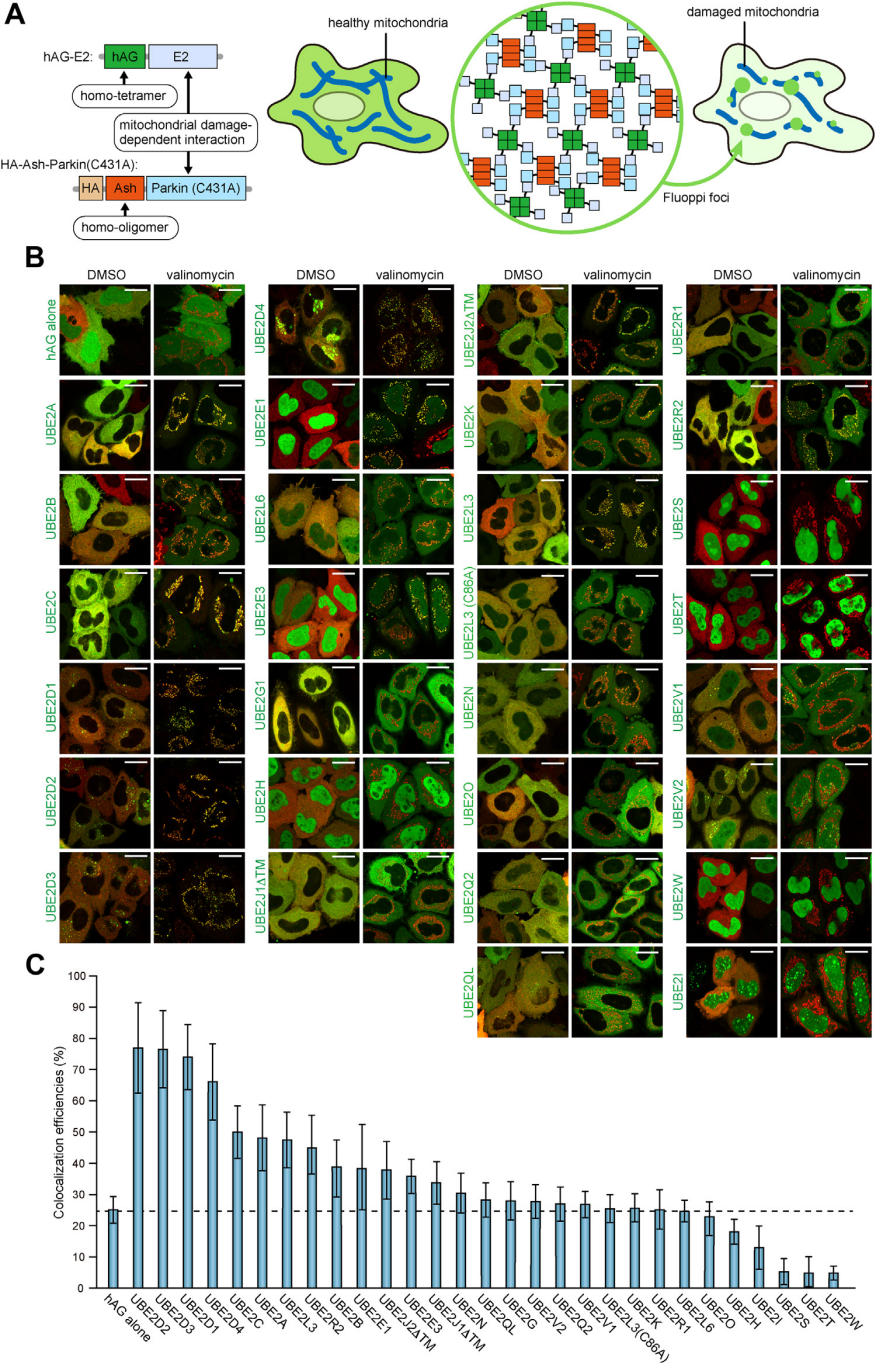
**Fluoppi analysis reveals intracellular interactions between various E2 enzymes and Parkin on dysfunctional mitochondria.***A*, schematic representation of Parkin–E2 Fluoppi. hAG-E2 forms a homotetramer, and HA-Ash-Parkin (C431A) forms a homooligomer. When mitochondrial damage induces the interaction between Parkin and E2, Fluoppi foci will be formed on the damaged mitochondria. *B*, HeLa cells expressing Ash-Parkin (C431A) (*red*) and the indicated hAG-tagged E2 enzymes (*green*) were treated with dimethyl sulfoxide (DMSO) or valinomycin for 3 h and then immunostained with an anti-Parkin antibody. Bars represent 10 μm. The same images are shown in Figs. S2 and S3 as merge along with the separated hAG-E2 and HA-Ash-Parkin images and TOMM20 signals. *C*, the efficiency of colocalization in (*A*) between various hAG-tagged E2 enzymes and TOMM20 after 3 h valinomycin treatment. Error bars represent mean ± SD of >50 cells counted in two independent experiments. Ash, Assembly helper; HA, hemagglutinin.

Taking advantage of the recruitment of Parkin to mitochondria, we next sought to identify the E2 enzymes interacting with Parkin on damaged mitochondria. For this purpose, we utilized fluorescence-based technology detecting PPIs (Fluoppi), which uses multivalent interactions to detect PPIs as fluorescent foci in cells (13, 19, 29) and has sufficient resolution to determine the subcellular localization of the PPI. To catalyze the multivalent interactions, homo-oligomeric Ash tag was fused to Parkin (C431A), and a homotetramer forming Azami-Green (hAG) tag was fused to various E2 enzymes. It is expected that, along the loss in mitochondrial membrane potential, Ash-Parkin (C431A) was recruited to damaged mitochondria where the REP region in Parkin is released from the core domain and hAG-tagged E2 starts to interact with Ash-Parkin (C431A) strongly. Therefore, through multivalent interactions composed of Ash and Ash, hAG and hAG, and Parkin and E2, Parkin (C431A) and E2 will form fluorescent foci on the mitochondria (Fig. 3*A*). We fused hAG tag to 30 different E2 enzymes and coexpressed them with Ash-Parkin (C431A) in HeLa cells that lack endogenous Parkin. Immunoblotting confirmed expression of the hAG-fusion proteins (Fig. S1), and under Tris(2-carboxyethyl)phosphine (TCEP) (thiol-free reducing agent)–treated conditions, most migrated as Ub-conjugated forms (Fig. S1). Under normal growth conditions, Ash-Parkin (C431A) and most of the hAG-tagged E2 enzymes were cytosolic (Figs. 3*B* and S2). UBE2D family proteins, including UBC2D1 (UbcH5a), UBE2D2 (UbcH5b), UBE2D3 (UbcH5c), and UBE2D4 (UbcH5d), localized as several dot-like structures in the cytosol. UBE2E3 (UbcH9) and UBE2H (UbcH2) mainly localized in the nucleus and had a weak cytosolic signal. UBE2E1 (UbcH6), UBE2I (Ubc9), UBE2S, UBE2T, and UBE2W (Ubc16) exclusively localized in the nucleus. Altered endoplasmic reticulum (ER) morphology was observed with the hAG-tagged forms of UBE2J1 and UBE2J2 since they are integrated into the ER membrane by a C-terminal transmembrane segment (Fig. S2). Therefore, we expressed mutants lacking the C-terminal transmembrane segment (UBE2J1ΔTM and UBE2J2ΔTM) to drive cytosolic localization (Figs. 3*B* and S2). Of note, no Ash-Parkin (C431A)-associated foci were observed under basal conditions with any of the hAG-E2 enzymes tested (Figs. 3*B* and S2), indicating self-inhibitory occlusion of the E2-binding site in the Ash-Parkin RING1 domain. Valinomycin treatment for 3 h induced Ash-Parkin (C431A) translocation to mitochondria, with many of the hAG-tagged E2 enzymes concomitantly recruited (Figs. 3*B* and S3). UBE2D1, UBE2D2, UBE2D3, and UBE2D4 were all efficiently recruited to mitochondria. UBE2L3, which has been reported to function as an E2 for Parkin but was not stably associated on mitochondria by GFP tagging (Fig. 1*D*), formed mitochondrial foci (Figs. 3*B* and S3), indicating that the Fluoppi method is capable of capturing E2–Parkin interactions in mitophagic cells. The mitochondrial recruitment of the hAG-tagged E2 enzymes was quantified in Figure 3*C* (see the detail in the *Experimental procedures* section). Interestingly, UBE2E1 was exclusively in the nucleus under basal conditions but was partially localized with Ash-Parkin to the mitochondria during mitophagy (Fig. 3*B*). UBE2C and UBE2A, which have not previously been reported to be E2s for Parkin, were also recruited to mitochondria. Conversely, although UBE2N was reported to contribute to Parkin translocation (24, 25), hAG-UBE2N formed few mitochondrial foci even when coexpressed with UBE2V1 and vice versa (Fig. 3*B* and S3). By incorporating the Fluoppi assay, we were able to monitor E2–Parkin interactions in live cells comprehensively and thus identify multiple E2 enzymes as new partners of Parkin during mitophagy.

### High correlation between Parkin–E2 interactions and mitochondrial ubiquitination

**Figure 4:**
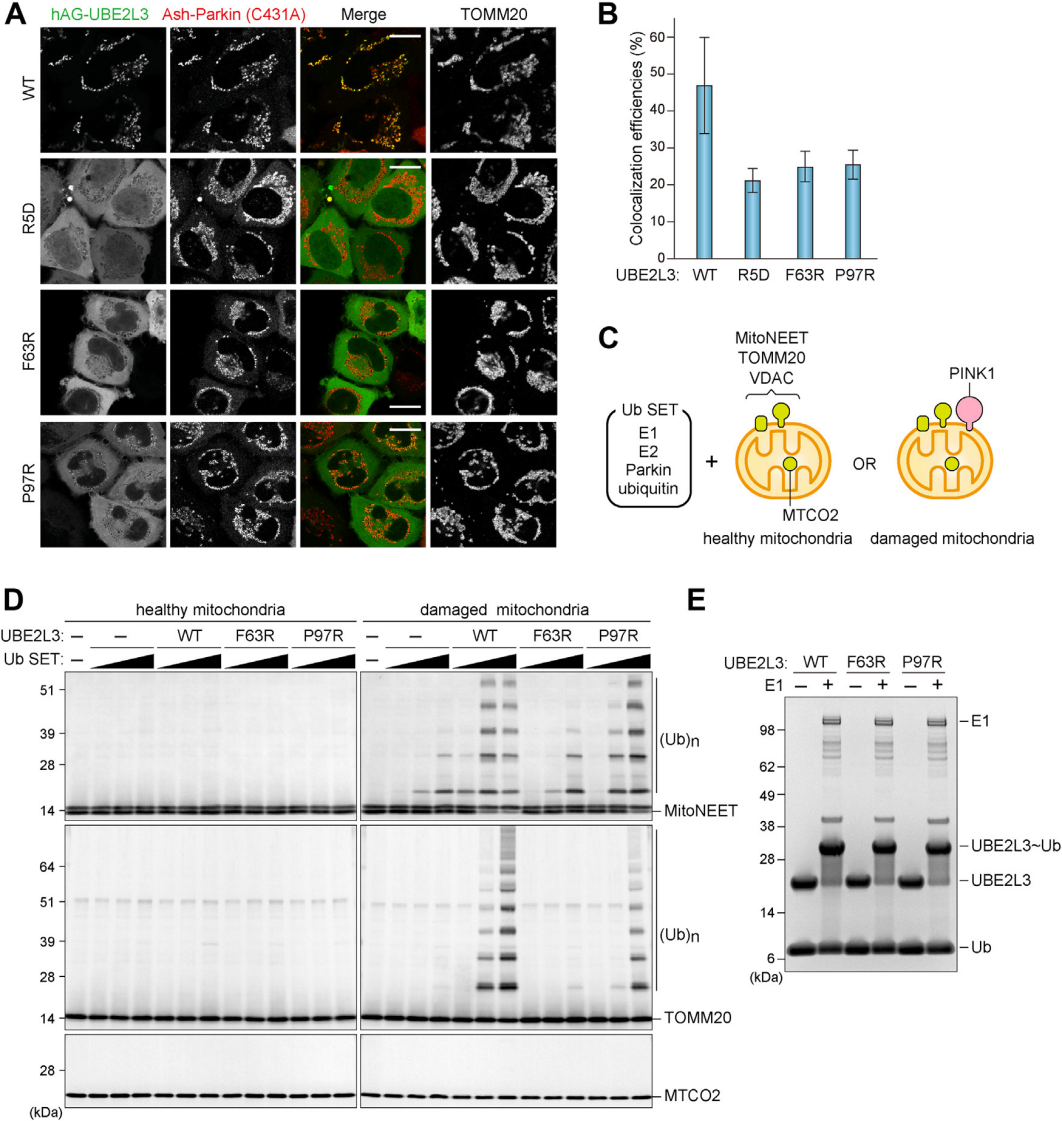
**UBE2L3 mutants defective for Parkin interactions do not mediate mitochondrial ubiquitination.***A*, HeLa cells expressing Ash-Parkin (C431A) and hAG-UBE2L3 (WT, R5D, F63R, or P97R) were treated with valinomycin for 3 h and then immunostained. Bars represent 10 μm. *B*, the efficiency of colocalization in (*A*) between the various hAG-UBE2L3 constructs and TOMM20 after 3 h valinomycin treatment. Error bars represent mean ± SD of >50 cells counted in two independent experiments. *C*, schematic diagram of *in vitro* mitochondrial ubiquitination. Ubiquitin (Ub) SET contains recombinant E1 (UBA1), E2, Parkin, and ubiquitin. Healthy mitochondria and damaged mitochondria were isolated from HeLa cells treated with dimethyl sulfoxide (DMSO) and valinomycin for 6 h, respectively. MitoNEET, TOMM20, and VDAC localize on the outer membrane, whereas MTCO2 is in the matrix. PINK1 accumulates only on the outer membrane of damaged mitochondria. *D*, healthy and damaged mitochondria were incubated with or without various concentrations of Ub SET at 32 °C for 30 min and immunoblotted with the indicated antibodies. (Ub)n denotes polyubiquitin chains. *E*, recombinant UBE2L3 was incubated with ubiquitin in the presence or the absence of E1 at 32 °C for 30 min. Samples were analyzed by SDS-PAGE under nonreducing conditions followed by Coomassie brilliant blue staining. Ash, Assembly helper.

UBE2L3 is known to mainly react with RBR E3s for the substrate ubiquitination. Based on the crystal structure of an activated Parkin and UBE2L3 complex, the N terminus and both loops 4 and 7 of UBE2L3 interact with the RING1 domain of Parkin (23). The side chains of Arg5 and Arg6 in UBE2L3 contribute to electrostatic interactions with an acidic region in the Parkin RING1 domain, and the highly conserved UBE2L3 residues Phe63 and Pro97 mediate contact with Parkin. We thus generated UBE2L3 mutants (R5D, F63R, or P97D) that are expected to disrupt interactions with Parkin and used the Fluoppi assay to assess their effects on mitochondrial recruitment. A loss in the mitochondrial membrane potential following valinomycin treatment for 3 h induced mitochondrial translocation of Ash-Parkin (C431A) as well as hAG-UBE2L3 WT. In contrast, no recruitment of the hAG-UBE2L3 R5D and F63R mutants was observed (Fig. 4*A*). Although the P97R mutant generated tiny fluorescent foci on mitochondria (Fig. 4*A*), quantification showed that the P97R mitochondrial recruitment defect is comparable to that of the R5D and F63R mutants (Fig. 4*B*). These results suggest that the UBE2L3 mutants are unable to physically interact with Parkin. Mitochondrial ubiquitination was also performed *in vitro* using recombinant UBE2L3 and Parkin. Mitochondria isolated from HeLa cells treated with dimethyl sulfoxide (healthy mitochondria) or valinomycin (damaged mitochondria) for 6 h were incubated with various concentrations of Ub SET, which contained recombinant E1, UBE2L3, Parkin, and Ub (Fig. 4*C*). No ubiquitination of the OMM proteins MitoNEET and TOMM20 was observed in healthy mitochondria (Fig. 4*D*), indicating that Parkin is not activated in healthy mitochondria. Conversely, robust ubiquitination of both MitoNEET and TOMM20 was detected on damaged mitochondria (Fig. 4*D*). No ubiquitination was observed for the mitochondrial matrix protein MTCO2 (Fig. 4*D*), confirming that Parkin accessibility is restricted to the OMM. Under these conditions, P97R partially and F63R greatly repressed mitochondrial ubiquitination (Fig. 4*D*). The recombinant UB2L3 R5D mutant was not assessed because of technical issues with bacterial overexpression. *In vitro* E2-conjugation assays revealed that the efficiency of loading Ub by the F63R and P97R mutants was comparable to that of the E1-dependent UBE2L3 WT (Fig. 4*E*). These results indicate a high correlation between Parkin–UBE2L3 interactions in cells and mitochondrial ubiquitination *in vitro*.

### Mitochondrial ubiquitination assays using various E2 enzymes

**Figure 5:**
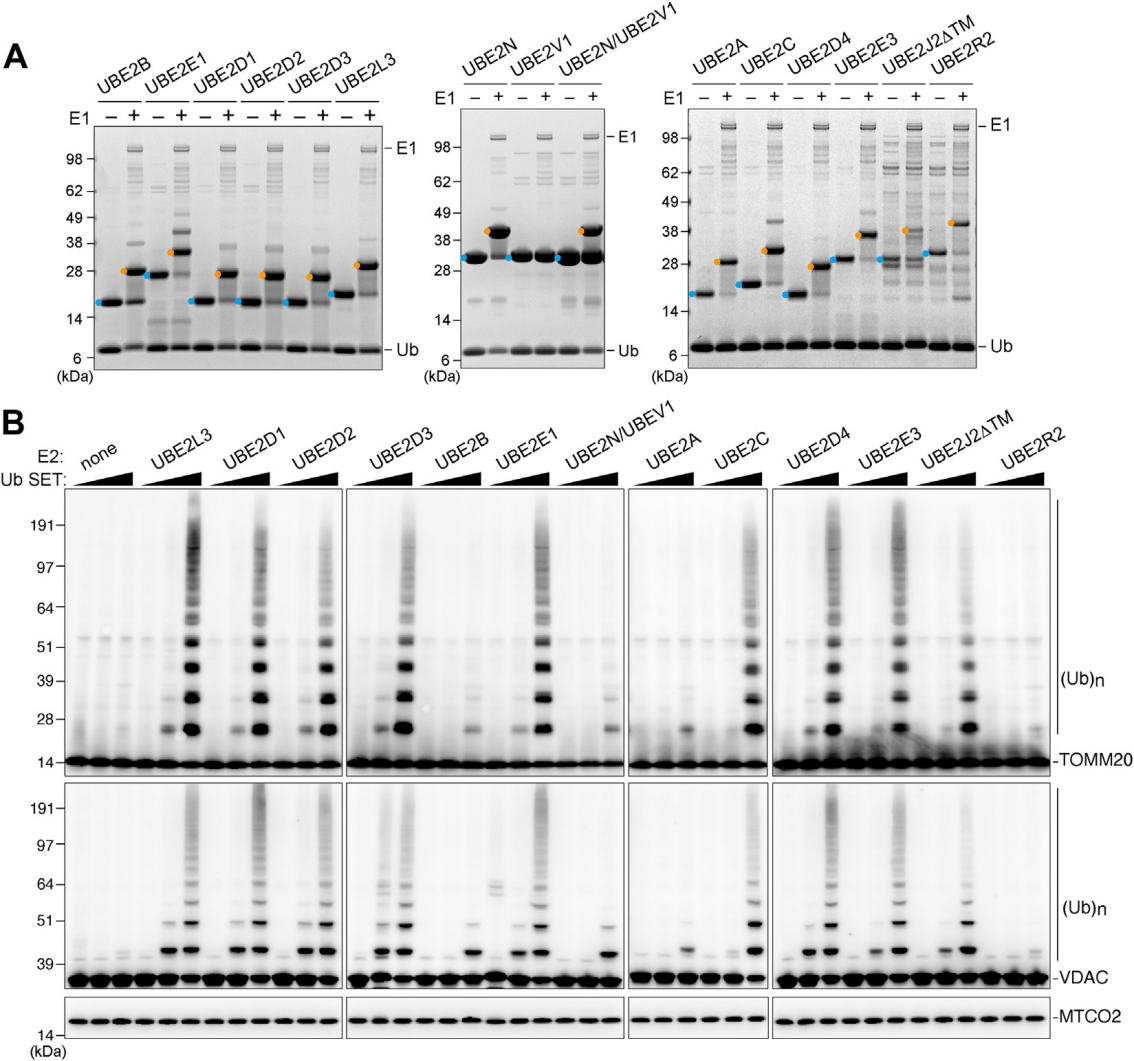
***In vitro* mitochondrial ubiquitination assay identifies E2s involved in Parkin activation.***A*, the indicated recombinant E2s were expressed in bacterial cells and purification using a nickel–nitrilotriacetic acid column. A ubiquitin (Ub) conjugation assay was performed by incubating the recombinant E2s and Ub with or without E1 at 32 °C for 30 min. Samples were analyzed by SDS-PAGE under nonreducing conditions followed by Coomassie brilliant *blue* staining. *Light blue* and *orange dots* denote E2 alone and Ub-conjugated E2, respectively. *B*, mitochondria isolated from valinomycin-treated HeLa cells were incubated with various concentrations of Ub SET (Fig. 4*C*) containing the indicated E2s at 32 °C for 30 min. Mitochondrial ubiquitination was analyzed by immunoblotting with anti-TOMM20, VDAC, and MTCO2 antibodies. (Ub)n denotes polyubiquitin chains.

We next sought to determine if the E2 enzymes identified in the Fluoppi as Parkin interaction partners could mediate mitochondrial ubiquitination. For this purpose, recombinant UBE2A, UBE2B, UBE2C, UBE2E1, UBE2E3, UBE2D1, UBE2D2, UBE2D3, UBE2D4, UBE2L3, UBE2J2ΔTM, and UBE2R2 were bacterially expressed and purified. Recombinant UBE2N and UBE2V1 were similarly expressed and purified. Apart from UBE2V1, the E2s tested were capable of conjugating Ub in an E1-dependent manner (Fig. 5*A*). *In vitro* mitochondrial ubiquitination assays using damaged mitochondria isolated from mammalian cultured cells and recombinant Ub SET showed that most of the E2 enzymes could ubiquitinate TOMM20 and VDAC in concert with Parkin (Fig. 5*B*). From these results, we classified UBE2L3, the UBE2D family, UBE2C, UBE2E1, UBE2E3, and UBE2J2 as high E2s. In contrast, UBE2A, UBE2B, UBE2N/UBE2V1, and UBE2R2 were classified as low E2s.

### Consensus sequence in E2 enzymes for Parkin interaction

**Figure 6:**
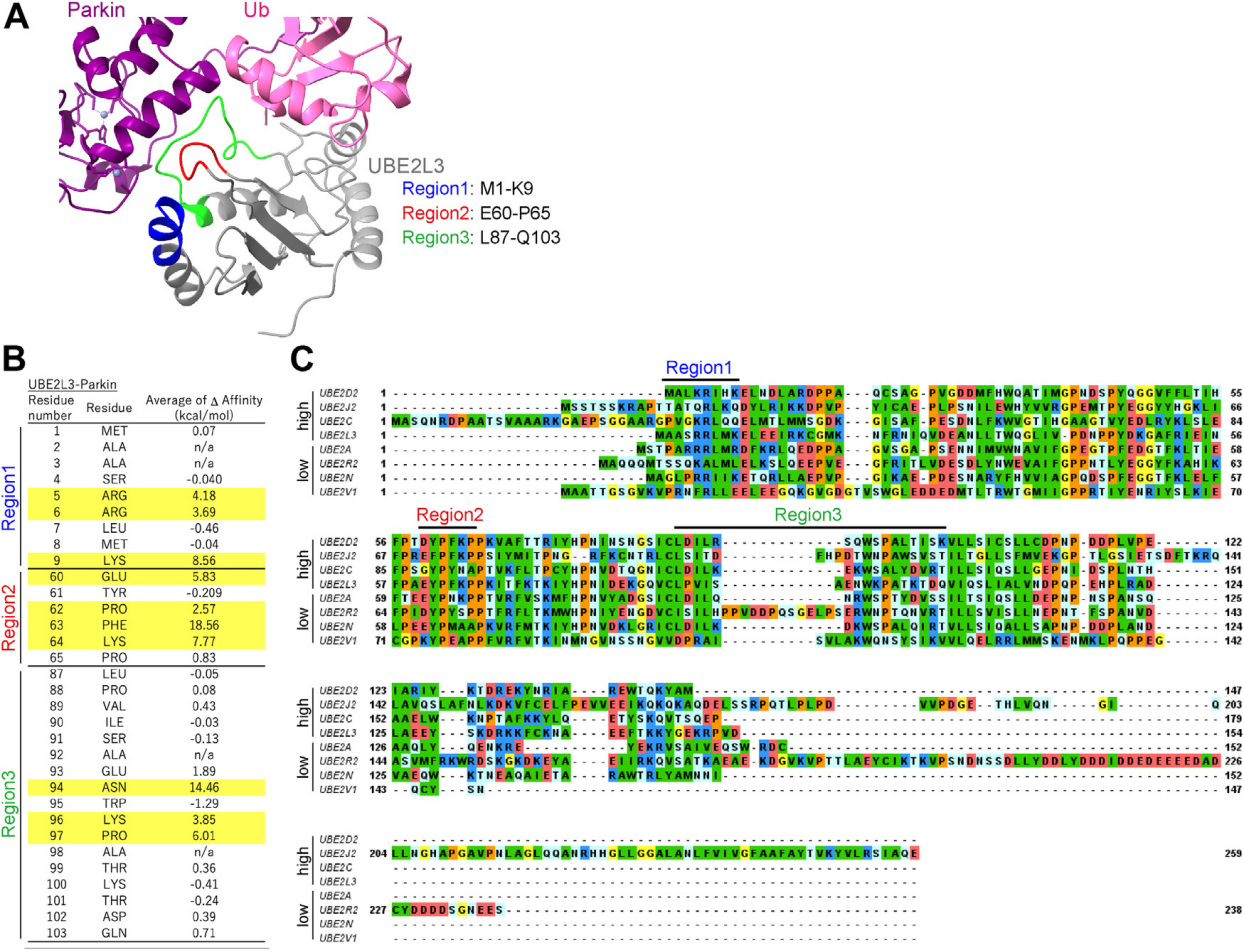
**Consensus sequence in E2s is critical for interacting with activated Parkin.***A*, three regions in UBE2L3 that are situated within 5 Å of Parkin were selected based on the UBE2L3–Parkin structure (Protein Data Bank ID: 6N13). *B*, changes in protein binding affinity (ΔAffinity) after Ala mutation of the target residue. Values were calculated using BioLuminate alanine scanning as implemented by Schrödinger Release 2021-4 (Schrödinger, LLC). *C*, multiple sequence alignment of E2s with high and low ubiquitination. The three Parkin proximal regions are indicated. The E2s having >60% sequence identity are removed.

The Fluoppi and *in vitro* ubiquitination assays identified many E2 enzymes that interact with Parkin to mediate ubiquitination of OMM proteins during mitophagy. Based on these findings, we sought to identify consensus sequences in E2 enzymes critical for interactions with activated Parkin. We estimated the consensus sequence based on amino acid conservation between predicted interaction hot spots in E2s with high ubiquitination activity and those with low ubiquitination activity. Using NMR-based structures of the UBE2L3–Parkin complex (Protein Data Bank ID: 6N13, 10 NMR structures) (30), we focused on three regions of UBE2L3 that had residues within 6 Å of Parkin (region 1: M1–K9, region 2: E60–P65, and region 3: L87–Q103) (Fig. 6*A*) and predicted the potential interaction hot spot residues based on differences in protein binding affinity (ΔAffinity) upon Ala substitution calculated using BioLuminate Ala scanning module (31) against the 10 NMR structures; those with average ΔAffinity >2.5 kcal/mol were defined as hot spot residues (Fig. 6*B*). We then assessed their degree of conservation between high and low ubiquitination E2s using multiple sequence alignment generated by Clustal Omega (32) (Fig. 6*C*). The E2 consensus sequence [D/E]xP[F/Y]KP (x is any residue) was estimated to reside between E60 and P65 in the high ubiquitination E2s.

### Fluoppi assay for interactions between UBE2L3 and other RBR-type E3 ligases

Although only 13 RBR-type E3s are encoded in human genomes, RBR-type E3s play important roles in various cellular functions and have been suggested to regulate their E3 activities *via* inter-PPIs or intra-PPIs. Therefore, we used the Fluoppi assay to gain insights into intracellular interactions between other RBR-type E3s and E2s. The linear Ub chain assembly complex (LUBAC) that consists of SHARPIN and two RBR-type E3 ligases, HOIP and HOIL-1L, facilitates NF- κB activation by generating linear Ub chains on substrates such as NEMO (33). HOIP is the catalytic center subunit in the LUBAC, and Cys885 in HOIP accepts Ub from UBE2L3 *via* a thioester bond (1, 4).

**Figure 7:**
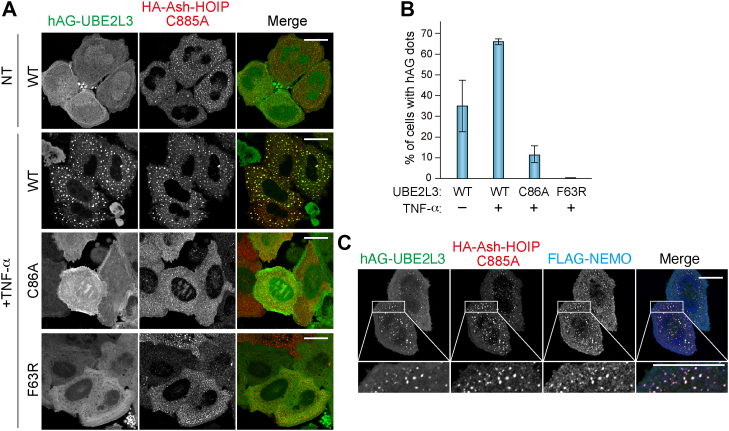
**Interactions between UBE2L3 and HOIP captured with the Fluoppi assay.***A*, HeLa cells expressing HA-Ash-HOIP (C885A) and hAG-UBE2L3 (WT, C86A, or F63R) were treated with dimethyl sulfoxide (DMSO) (NT) or tumor necrosis factor alpha (TNF-α) for 10 min and then immunostained with an anti-HA antibody. Bars represent 10 μm. *B*, the percentage of cells in (*A*) with foci consisting of HA-Ash-HOIP and hAG-UBE2L3. Error bars represent mean ± SD of >100 cells counted in each of three independent experiments. *C*, HeLa cells expressing HA-Ash-HOIP (C885A), hAG-UBE2L3, and FLAG-NEMO were treated with TNF-α for 10 min and then immunostained with anti-HA and anti-FLAG antibodies. *Insets* represent magnified images of the areas indicated. Bars represent 10 μm. Ash, Assembly helper; HA, hemagglutinin.

HeLa cells were transfected with HA-Ash-HOIP (C885A) and hAG-UBE2L3 and then treated with proinflammatory cytokine tumor necrosis factor alpha (TNF-α). As shown in Figure 7*A*, HOIP (C885A) formed dot-like structures and hAG-UBE2L3 WT localized to the foci. The efficiencies of hAG-UBE2L3 foci formation were higher than those without TNF-α treatment (Fig. 7, *A* and *B*). In contrast, when expressing the UBE2L3 C86A or F63R mutants, the size of the HOIP (C885A) foci was much smaller than those of UBE2L3 WT, and neither UBE2L3 C86A nor F63R was recruited to the foci (Fig. 7, *A* and *B*). The lack of hAG-UBE2L3 C86A foci suggests that the Ub conjugated to UBE2L3 is required for E2–LUBAC interactions, which is consistent with structural analyses (34). Impaired foci formation in the UBE2L3 F63R mutant indicates that the interaction surface on UBE2L3 for Parkin is also used for HOIP interactions. Furthermore, NEMO, a LUBAC substrate, was recruited to the foci (Fig. 7*C*), indicating that the Fluoppi foci are active sites for LUBAC assembly and substrate ubiquitination.

**Figure 8:**
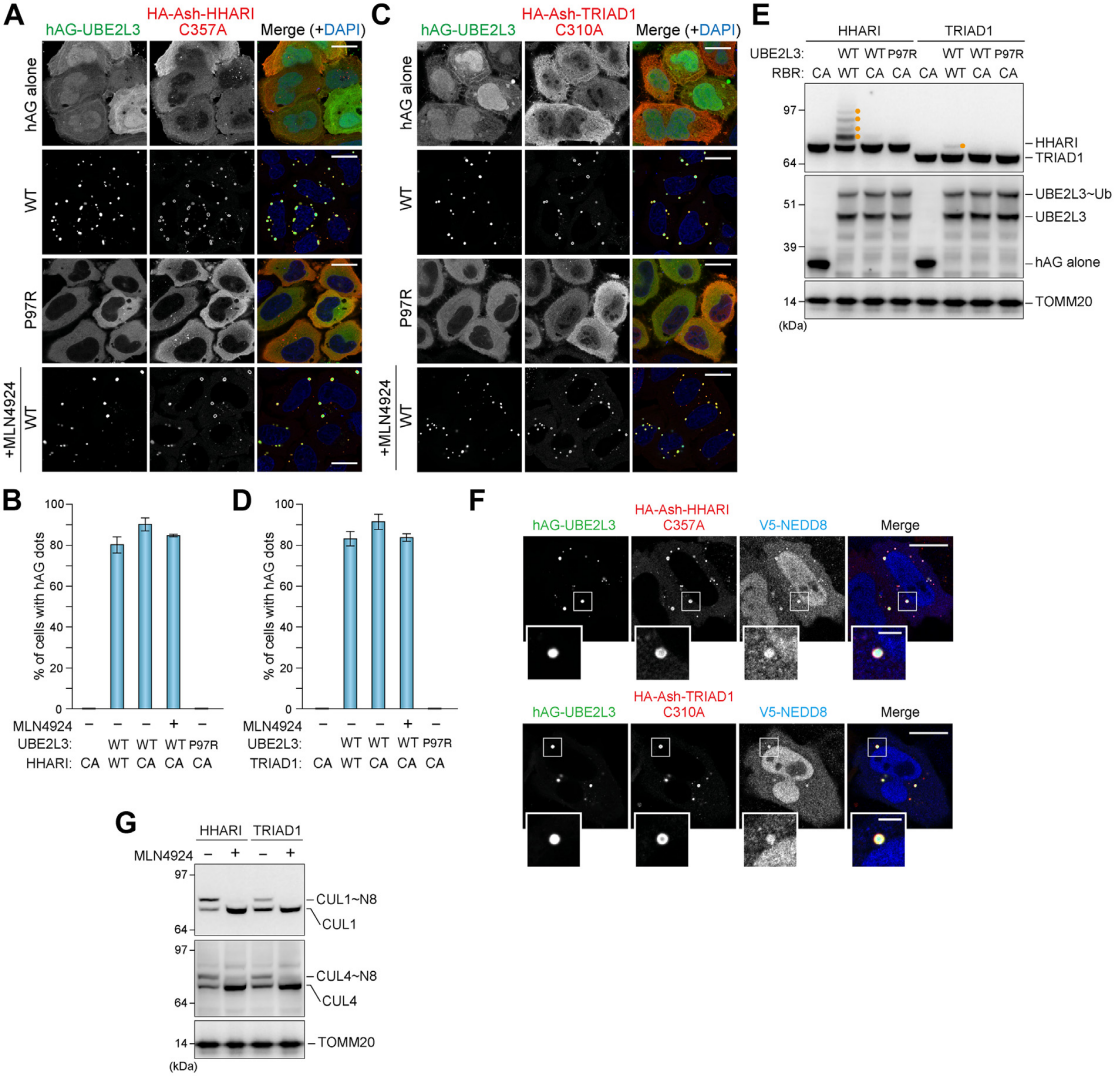
**HHARI and TRIAD1 interactions captured with the Fluoppi assay.***A*, HeLa cells expressing hAG constructs (hAG alone, hAG-UBE2L3 WT, or P97R) and HA-Ash-HHARI C357A were immunostained with an anti-HA antibody. To inhibit neddylation, cells were treated overnight with 1 μM MLN4924 and then immunostained. Nuclei were stained with 4′,6-diamidino-2-phenylindole (DAPI). Bars represent 10 μm. *B*, the percentage of cells in (*A*) with foci. CA denotes the HHARI C357A mutant. Error bars represent mean ± SD of >100 cells counted in each of three independent experiments. *C*, HeLa cells expressing the hAG constructs and HA-Ash-TRIAD1 C310A were immunostained with an anti-HA antibody. Bars represent 10 μm. *D*, the percentage of cells in (*C*) with foci. CA denotes the TRIAD1 C310A mutant. Error bars represent mean ± SD of >100 cells counted in each of three independent experiments. *E*, total cell lysates prepared from HeLa cells expressing hAG constructs (hAG alone or hAG-UBE2L3) and HA-Ash-RBR constructs (HHARI or TRIAD1) were immunoblotted with anti-HA, anti-AG, and anti-TOMM20 antibodies. *Orange dots* represent ubiquitinated HHARI and TRIAD1. *F*, HeLa cells expressing hAG-UBE2L3, V5-NEDD8, and HA-Ash-HHARI C357A or HA-Ash-TRIAD1 C310A were immunostained with anti-HA and V5 antibodies. Bars represent 10 μm; bars in magnification panels represent 2 μm. *G*, inhibition of neddylation was confirmed by immunoblotting. HeLa cells expressing HA-Ash-HHARI or HA-Ash-TRIAD1 were treated with or without MLN4924 overnight. Total cell lysates were immunoblotted with anti-CUL1, anti-CUL4, and anti-TOMM20 antibodies. Ash, Assembly helper; HA, hemagglutinin.

We next examined to other RBR-type E3s—HHARI (ARIH1) and TRIAD1 (ARIH2). HHARI and TRIAD1 bind neddylated Cullin–RING ligase complexes and prime the selected substrate with monoubiquitin, which accelerates subsequent polyubiquitination by the Cullin–RING ligase (35, 36). Both HHARI and TRIAD1 are ARIH family proteins that contain an Ariadne domain. Because previous studies were largely *in vitro* based, the intracellular localization of HHARI and TRIAD1 activity remains unknown. When we expressed the catalytic mutant HA-Ash-HHARI (C357A) with hAG, it was diffusely localized in both the cytosol and nucleus, indicating that HHARI (C357A) does not interact with hAG (Fig. 8*A*). Conversely, exclusively cytosolic Fluoppi foci were observed when HA-Ash-HHARI (C357A) and hAG-UBE2L3 WT were coexpressed (Fig. 8, *A* and *B*). HA-Ash-HHARI appeared to localize only outer surface of the foci, but it is because anti-HA antibody cannot access the interior of Fluoppi condensates. Importantly, no stimulus, such as valinomycin for Parkin or TNF-α for LUBAC, was required for the interaction between HHARI and UBE2L3. A similar Fluoppi-based interaction was detected for TRIAD1 and UBE2L3 (Fig. 8, *C* and *D*). Although both HHARI and TRIAD1 exhibit autoinhibition (*i.e.*, the Ariadne domain occludes the RING2 domain catalytic Cys site), the RING1 domain can bind UBE2L3 regardless of the Ariadne domain (3). Immunoblots showed that autopolyubiquitinated HHARI and automonoubiquitinated TRIAD1 were only detected with the ligase-active WT form but not for catalytic inactive mutants (Fig. 8*E*). Next, we examined if neddylation impacts the HHARI–UBE2L3 and TRIAD1–UBE2L3 interactions since both are reported to be allosterically activated by a neddylated Cullin–RING ligase (35), and covalent linkage of NEDD8 to Cullin can activate Cullin–RING ligase-mediated ubiquitination (37). When we expressed V5-tagged NEDD8 (V5-NEDD8) with hAG-UBE2L3 WT and Ash-HHARI (C357A) or Ash-TRIAD1 (C310A), V5-NEDD8 was detected on the foci (Fig. 8*F*). However, neither the TRIAD1–UBE2L3 interaction nor the HHARI–UBE2L3 interaction is NEDD8 dependent as the neddylation inhibitor (MLN4924) had no effect on foci formation (Fig. 8, *A*–*D*), whereas CUL1 and CUL4, both of which are neddylated, completely disappeared (Fig. 8*G*).

## Discussion

Many RBR-type E3s exhibit autoinhibition, which restricts their ubiquitination function to specific conditions. For Parkin activation, a PINK1 phosphorylation–dependent conformational change that occurs in response to mitochondrial damage removes occlusion of the E2-binding site and catalytic Cys residue by the REP and RING0 domains (22, 38, 39). Furthermore, although physical interactions between E2 enzymes and activated Parkin are required for mitochondrial ubiquitination, the transience of the E2–E3 interaction, which could not be directly detected in live cells, limited their identification. In this study, we developed a Fluoppi-based method that overcomes this limitation and demonstrated that UBE2L3 and UBE2D family proteins as well as UBE2E1 and UBE2J2 interact with Parkin on damaged mitochondria. We further verified these interactions *via in vitro* ubiquitination assays. Fluoppi is a genetically encoded PPI visualization system that allows us to see cellular protein interactions as fluorescent foci. Fluoppi is very easy to be applied, and a sensitive PPI-detection method as compared with other systems such as FRET since Fluoppi-based PPIs maintain even after cell fixation as well as intracellular localization where PPIs occurs is also obtained (29). However, two proteins fused with hAG or Ash should not be membrane proteins. As we examined in this study, when membrane-anchored E2 enzymes UBE2J1 and UBE2J2 were fused with hAG, altered ER morphology was observed (Fig. S2). Therefore, protein interactions with membrane proteins had better to be examined by other PPI techniques.

The UBE2N–UBE2V1 complex is known to create K63-linked chains (40), and Parkin-mediated mitophagy induces massive K63-linked chain production on damaged mitochondria (16). It, however, was not among the E2s identified in our in-cell Fluoppi assay or the *in vitro* assay, which function in Parkin-mediated mitochondrial ubiquitination. This is consistent with Ordureau *et al.* (16), who showed using an *in vitro* Parkin autoubiquitination assay that Parkin generates K63-linked chains with the help of various E2s including UBE2L3, but not the UBE2N–UBE2V1 complex. However, this does not preclude the possibility that the complex elongates K63 chains as a branched Ub chain that is derived from a Parkin-mediated ubiquitination seed on the OMM catalyzed (41). Our results also indicate that ER-resident UBE2J2 acts with Parkin. McLelland *et al.* (42) reported that Parkin activation at contact points between the ER and mitochondria results in Mfn2 ubiquitination and subsequent dissociation of contact between the organelles. Therefore, UBE2J2 may be required for local activation of Parkin that is near the ER.

Based on an X-ray structure of phosphorylated Parkin in complex with UBE2L3 and pUb, the binding of the phosphorylated UBL domain to the RING1 domain results in dissociation of the RING2 domain from the core (23, 30). Condos *et al.* (30) presented a model of UBE2L3∼Ub bound to activated Parkin based on the NMR chemical shift analysis and found that the conjugated Ub associates with the RING1–IBR interface in Parkin. Our Fluoppi assay with the UBE2L3 C86A mutation indicated that UBE2L3–Parkin interaction is reduced when Ub cannot be conjugated to UBE2L3 (Fig. 3). These results strongly suggest that Ub conjugated on E2 stabilizes the Parkin–E2 complex. Furthermore, by coupling in-cell and *in vitro* analyses with bioinformatics, we identified a weak, but more accurate than previously reported (23), consensus sequence that mediates E2 interactions with Parkin.

In this study, we also examined the interaction between UBE2L3 and the LUBAC complex. The Fluoppi foci consisting of UBE2L3 and HOIP facilitated proinflammatory cytokine TNF-α. Previously, a structure of the active HOIP–E2∼Ub complex revealed that the RING1–IBR module forms an elongated arm to capture Ub molecules conjugated to UBE2D2 (UbcH5B) (34). Therefore, when LUBAC is activated, HOIP simultaneously recognizes both E2 and Ub conjugated to the E2. The interaction between UBE2L3 and ARIH family E3s was also tested. The fundamental and initial requirement of E3 ligase function is to bind to E2s. In the case of Parkin, UBE2L3 binding ability is coupled with E3 ligase activity. UBL phosphorylation leads to a conformation change in Parkin that exposes both the E2 binding site and catalytic Cys in a sequential manner. In sharp contrast, HHARI and TRIAD1 allow UBE2L3 binding under basal conditions but are E3 inactive because release of the masked catalytic Cys in RING2 by the Ariadne domain is not coupled to E2 binding (43). Our in cell Fluoppi assay results are consistent with previous *in vitro* studies.

Our understanding of RBR-type E3s is limited with many currently understudied. RNF144A and RNF144B (IBRDC2) contain a transmembrane segment at their C terminus that regulates E3 activity (44). RNF144A is thought to promote apoptosis (45), and RNF144B has been suggested to regulate both apoptosis (46) and inflammasome responses (47). However, no definitive substrates ubiquitinated by the proteins have been identified, and intracellular localization of the enzymatically active forms remains to be determined. Although RNF19A has been reported to prevent neurotoxicity, the mechanism is currently unknown (48). Similarly, although RNF216 (TRIAD3), another RBR-type E3, has been linked to the neurodegenerative disorder Gordon–Holmes syndrome (49) and its activity is enhanced by phosphorylation and K63-linked di-Ub (50), neither the subcellular localization of the activated protein nor its substrates are known. Our Fluoppi method, however, can identify *bona fide* E2–RBR-type E3 pairings as well as provide intracellular localization of RBR-type E3 activation. As such, this method is an ideal tool for investigating RBR-type E3s and their substrates.

## Experimental procedures

### Plasmids

**Table:** 

Plasmid DNA	Source
pMXs-puro_GST-Parkin	This study
pEGFP-C1_UBE2L3 (GFP-UBE2L3)	This study
mCherry-Parkin	Yamano *et al.*, *Elife* (52)
pEGFP-C1_Parkin	Matsuda *et al.*, *JCB* (10)
pEGFP-C1_Parkin C431A	Iguchi *et al.*, *JBC* (53)
HA-Parkin	Matsuda *et al.*, *JCB* (10)
HA-Parkin C431A	Iguchi *et al.*, *JBC* (53)
Ash-Parkin	This study
Ash-Parkin C431A	Yamano *et al.*, *JBC* (19)
Ash-Parkin A320R/C431A	This study
pMXs-puro_GST-Parkin C431A	This study
pMXs-puro_GST-Parkin A320R/C431A	This study
phAG-MCL	MBL (AM-8011M)
phAG-UBE2A	This study
phAG-UBE2B	This study
phAG-UBE2C	This study
phAG-UBE2D1	This study
phAG-UBE2D2	This study
phAG-UBE2D3	This study
phAG-UBE2D4	This study
phAG-UBE2E1	This study
phAG-UBE2L6	This study
phAG-UBE2E3	This study
phAG-UBE2G1	This study
phAG-UBE2H	This study
phAG-UBE2J1	This study
phAG-UBE2J1ΔTM	This study
phAG-UBE2J2	This study
phAG-UBE2J2ΔTM	This study
phAG-UBE2K	This study
phAG-UBE2L3	This study
phAG-UBE2L3 C86A	This study
phAG-UBE2N	This study
phAG-UBE2O	This study
phAG-UBE2Q2	This study
phAG-UBE2QL	This study
phAG-UBE2R1	This study
phAG-UBE2R2	This study
phAG-UBE2S	This study
phAG-UBE2T	This study
phAG-UBE2V1	This study
phAG-UBE2V2	This study
phAG-UBE2W	This study
phAG-UBE2I	This study
phAG-UBE2L3 R5D	This study
phAG-UBE2L3 F63R	This study
phAG-UBE2L3 P97R	This study
HA-Ash-HOIP C885A	This study
FLAG-NEMO	A gift from Oikawa and Tokunaga
HA-Ash-HHARI WT	This study
HA-Ash-HHARI C357A	This study
HA-Ash-TRIAD1 WT	This study
HA-Ash-TRIAD1 C310A	This study
V5-NEDD8	This study

### Antibodies

**Table:** 

Reagent or resource	Source	Identifier
Antibodies for immunoblotting		
Mouse monoclonal anti-HA (TANA2)	MBL	Catalog no.: M180-3, Research Resource Identifier (RRID): AB_10951811
Rabbit polyclonal anti-TOMM20 (FL-145)	Santa Cruz Biotechnologies	Catalog no.: sc-11415, RRID: AB_2207533
Mouse monoclonal anti-MTCO2 (12C4F12)	Abcam	Catalog no.: Ab110258
Rabbit polyclonal anti-Azami-Green	MBL	Catalog no.: PM011M
Rabbit polyclonal anti-MitoNEET (CISD1)	Proteintech	Catalog no.: 16006-1-AP, RRID: AB_2080268
Mouse monoclonal anti-VDAC (89-173/025)	Calbiochem	Catalog no.: 529534
Mouse monoclonal anti-CUL-1 (D-5)	Santa Cruz Biotechnologies	Catalog no.: sc-17775, RRID: AB_627325
Goat polyclonal anti-CUL-4 (C-19)	Santa Cruz Biotechnologies	Catalog no.: sc-8557, RRID: AB_2261178
Mouse monoclonal anti-Parkin (PRK8)	Millipore	Catalog no.: MAB5512, RRID: AB_2267915
Goat anti-rabbit IgG horseradish peroxidase linked	Jackson ImmunoResearch	Catalog no.: 111-035-144
Antimouse IgG horseradish peroxidase linked	Promega	Catalog no.: W402B
Antibodies for immunostaining		
Rabbit polyclonal anti-TOMM20 (FL-145)	Santa Cruz Biotechnologies	Catalog no.: sc-11415, RRID: AB_2207533
Mouse monoclonal anti-Parkin (PRK8)	Santa Cruz Biotechnologies	Catalog no.: sc-32282, RRID: AB_628104
Mouse monoclonal antimultiubiquitin (FK2)	MBL	Catalog no.: D058-3, RRID: AB_592937
Rabbit polyclonal anti-phos-Ubiquitin (pS65)	Okatsu *et al.*	Not applicable
Rabbit polyclonal anti-Azami-Green	MBL	Catalog no.: PM011M
Mouse monoclonal anti-HA (TANA2)	MBL	Catalog no.: M180-3, RRID: AB_10951811
Rat monoclonal anti-HA (3F10)	Roche	Catalog no.: 11867423001, RRID: AB_390918
Mouse monoclonal anti-DDDDK (FLA-1)	MBL	Catalog no.: M185-3L, RRID: AB_11123930
Mouse monoclonal anti-V5 tag	Thermo Fisher Scientific	Catalog no.: R960-25, RRID: AB_2556564
Goat anti-rabbit IgG Alexa Fluor 488 conjugated	Invitrogen	Catalog no.: A-11034
Goat antimouse IgG Alexa Fluor 488 conjugated	Invitrogen	Catalog no.: A-11029
Goat anti-rabbit IgG Alexa Fluor 568 conjugated	Invitrogen	Catalog no.: A-11036
Goat antimouse IgG Alexa Fluor 568 conjugated	Invitrogen	Catalog no.: A-11031
Goat anti-rabbit IgG Alexa Fluor 647 conjugated	Invitrogen	Catalog no.: A-21245
Goat antimouse IgG Alexa Fluor 647 conjugated	Invitrogen	Catalog no.: A-21236
Goat antirat IgG Alexa Fluor 488 conjugated	Invitrogen	Catalog no.: A-11006
Goat antirat IgG Alexa Fluor 568 conjugated	Invitrogen	Catalog no.: A-11077
Goat antirat IgG Alexa Fluor 647 conjugated	Invitrogen	Catalog no.: A-21247

### Cells and transfections

HeLa cells were cultured in Dulbecco's modified Eagle's medium (Sigma–Aldrich) supplemented with 10% (v/v) fetal bovine serum (Biowest), 1 mM sodium pyruvate (Gibco), nonessential amino acids (Gibco), and penicillin–streptomycin–glutamine (Gibco) at 37 °C in a 5% CO_2_ incubator. FuGENE6 transfection reagent (Promega) was used for transient expression according to the manufacturer’s instructions.

Valinomycin (Sigma–Aldrich), TNF-α (Promega), and MLN4924 (neddylation inhibitor; Abcam) were used at final concentrations of 10 μM, 10 ng/ml, and 1 μM, respectively.

### Preparation of recombinant proteins

Recombinant GST-rat Parkin and His6-human ubiquitin were described previously (19). Recombinant E1 was prepared as follows. *Escherichia coli* BL21-CodonPlus(DE3)-RIL (Agilent Technologies) cells harboring pGEX6P1_GST-human E1(UBA1)-His8 were grown in LB medium supplemented with 100 μg/ml ampicillin and 25 μg/ml chloramphenicol at 37 °C. GST-E1-His8 was overexpressed at 16 °C for 16 h by the addition of 0.3 mM IPTG. The bacterial cell pellets after centrifugation were resuspended in Tris-buffered saline (TBS) (50 mM Tris–HCl [pH 7.5] and 120 mM NaCl) supplemented with DNase I (Worthington), MgCl_2_, lysozyme (Wako), DTT, and protease inhibitor cocktail (Roche) and frozen using liquid nitrogen. The frozen cell suspension was thawed, sonicated, and insoluble proteins including cell debris were removed by centrifugation and a Millex filter (Millipore). The supernatant was mixed with equilibrated glutathione-Sepharose 4B (GE Healthcare) for 30 min at 4 °C. The sepharose was loaded onto a column and then washed with TBS containing 1 mM TCEP (Sigma–Aldrich). Proteins eluted with TBS containing 1 mM TCEP and 20 mM reduced l-glutathione (Sigma–Aldrich) were then mixed with nickel–nitrilotriacetic acid agarose (Qiagen) for 30 min at 4 °C. The bound proteins were eluted with imidazole, and recombinant GST-E1-His8 was suspended in TBS containing 1 mM TCEP and 10% (w/v) glycerol *via* a PD Miditrap G-25 (GE Healthcare) and concentrated using Amicon Ultra centrifugal filters (Millipore). Recombinant E2 enzymes were prepared as follows. *E. coli* BL21-CodonPlus(DE3)-RIL cells harboring pT7-7_His6-tagged human E2s (UBE2A, UBE2B, UBE2C, UBE2D1, UBE2D2, UBE2D3, UBE2D4, UBE2E1, UBE2E3, UBE2N, UBE2V1, UBE2J2ΔTM, UBE2R2, UBE2L3 WT, F63R, or P97R) were grown in LB medium supplemented with 100 μg/ml ampicillin and 25 μg/ml chloramphenicol at 37 °C. IPTG induction and cell lysis protocols were as described previously. The supernatant was mixed with equilibrated nickel–nitrilotriacetic acid agarose for 30 min at 4 °C. The agarose was loaded onto a column and washed with TBS containing 1 mM TCEP (Sigma–Aldrich). The proteins were eluted with TBS containing 1 mM TCEP and 200 mM imidazole, and recombinant His6-E2s were suspended in TBS containing 1 mM TCEP and 10% (w/v) glycerol.

### *In vitro* mitochondrial ubiquitination assay

Mitochondria were isolated from HeLa cells treated for 6 h with dimethyl sulfoxide (for healthy mitochondria) or valinomycin (for damaged mitochondria). HeLa cells grown on 24 × 15 cm dishes were collected, washed with PBS twice, and homogenized using a 25G needle and syringe in solution A (20 mM Hepes–KOH [pH 7.6], 220 mM mannitol, 70 mM sucrose, 1 mM EDTA, 0.5 mM PMSF [Nacalai Tesque], and 2 mg/ml fatty acid–free bovine serum albumin [Sigma–Aldrich]). Cell homogenates were centrifugated at 800*g* at 4 °C for 10 min. The supernatants were then centrifuged at 10,000*g* at 4 °C for 20 min. The pellets were resuspended in sucrose buffer (10 mM Hepes–KOH [pH 7.6] and 500 mM sucrose) and stored at −80 °C until used. The final concentration of the Ub SET, which consisted of recombinant GST-E1-His8 (75 nM), His6-E2 (3 μM), GST-Parkin (1.5 μM), and His6-Ub (15 μM), was mixed with 5 mg/ml isolated mitochondria in reaction buffer (10 mM Hepes–KOH [pH 7.6], 220 mannitol, 70 mM sucrose, 60 mM NaCl, 0.5 mM TCEP, 2.5 mM ATP, and 5 mM MgCl_2_) for 30 min at 32 °C. Ub SET diluted 10- or 100-fold were similarly incubated. Mitochondria were washed with solution B (20 mM Hepes–KOH [pH 7.6], 220 mM mannitol, 70 mM sucrose, and 1 mM EDTA) and lysed with SDS-PAGE sample buffer.

### E2-conjugation assay

Recombinant 0.385 μM GST-E1-His8, 15 μM His6-E2, and 22.5 μM His6-Ub were incubated in conjugation buffer (10 mM Hepes–KOH [pH 7.6], 60 mM NaCl, 5 mM ATP, 10 mM MgCl_2_, and 1 mM TCEP) for 1 h at 32 °C. SDS-PAGE sample buffer without DTT was then added to the samples. The proteins were separated by SDS-PAGE using NuPAGE 4 to 12% Bis–Tris gels (Invitrogen) and visualized by Coomassie brilliant blue staining (Coomassie brilliant blue Stain One; Nacalai Tesque).

### Immunoblotting

Cells grown on 6-well plates were washed twice with PBS, lysed with appropriate volumes of SDS-PAGE sample buffer supplemented with either TCEP or DTT, sonicated, and then boiled at 95 °C for 5 min. Total cell lysates were loaded on NuPAGE 4 to 12% Bis–Tris gels and electrophoresed using Mes or Mops running buffer according to the manufacturer’s instructions. For analysis of Parkin phosphorylation, total cell lysates were loaded on Tris–glycine polyacrylamide gels containing 50 μM Phos-tag (Wako) and 100 μM MnCl_2_. Proteins were transferred to polyvinylidene difluoride membranes. The membranes were blocked with 2% (w/v) skim-milk in TBS with Tween-20 buffer, incubated with primary antibodies, washed with TBS with Tween-20, and incubated with horseradish peroxidase–conjugated secondary antibodies. Proteins were detected using a Western Lightning Plus-ECL Kit (PerkinElmer) and an ImageQuant LAS 4000 imaging system (GE Healthcare).

### Immunocytochemistry

Cells grown on glass bottom 35-mm dishes were fixed with 4% paraformaldehyde in PBS for 25 min. The cells were permeabilized with 0.15% (v/v) Triton X-100 in PBS for 20 min, preincubated with 0.1 (w/v) gelatin in PBS for 30 min, and then incubated with primary antibodies. After washing with PBS with Tween-20, the cells were incubated with Alexa Fluor–conjugated secondary antibodies. Microscopy images were captured using an inverted confocal microscope (LSM780; Carl Zeiss) with a Plan-Apochromat 63×/1.4 Oil differential interference contrast lens. Image analysis was performed with ZEN microscope software and Photoshop (Adobe). ZEN software was used for colocalization analysis. Individual cells were initially selected as regions of interest with the appropriate hAG and TOMM20 signal thresholds set in each cell. Overlapping hAG and TOMM20 signals per total hAG signal intensity in a single region of interest (weighted colocalization coefficients) were determined.

## Data availability

The data generated are included in the main test file and supporting information.

## Supporting information

This article contains supporting information.

## Conflict of interest

The authors declare that they have no conflicts of interest with the contents of this article.

## Supporting information

Supplemental Figure S1 **Ubiquitin conjugation of hAG-tagged E2 enzymes.** Total cell lysates prepared from HeLa cells expressing the indicated hAG-tagged E2s were electrophoresed in non-reducing (TECP) or reducing (DTT) conditions and then immunoblotted with an anti-Azami Green antibody. Light blue and orange dots denote E2 alone and Ub-conjugated E2, respectively.

Supplemental Figure S2 **Intracellular localization of hAG-tagged E2s under basal conditions.** HeLa cells expressing Ash-Parkin (C431A) and the indicated hAG-tagged E2 enzymes were treated for 3 hrs with DMSO and then immunostained with anti-Parkin and TOMM20 antibodies. Bars, 10 μm. The same merge images are shown in Figure 3*B*.

Supplemental Figure S3 **Intracellular localization of hAG-tagged E2s under mitophagy conditions.** HeLa cells expressing Ash-Parkin (C431A) and the indicated hAG-tagged E2 enzymes were treated for 3 hrs with valinomycin and then immunostained with anti-Parkin and TOMM20 antibodies. Bars, 10 μm. The same merge images are shown in Figure 3*B*.
