# Supplementary figures and images for: Elucidation of ubiquitin-conjugating enzymes that interact with RBR-type ubiquitin ligases using a liquid–liquid phase separation–based method
Source: J Biol Chem. 2022 Dec 21;299(2):102822. doi: 10.1016/j.jbc.2022.102822 (PMC9860496; doi:10.1016/j.jbc.2022.102822)

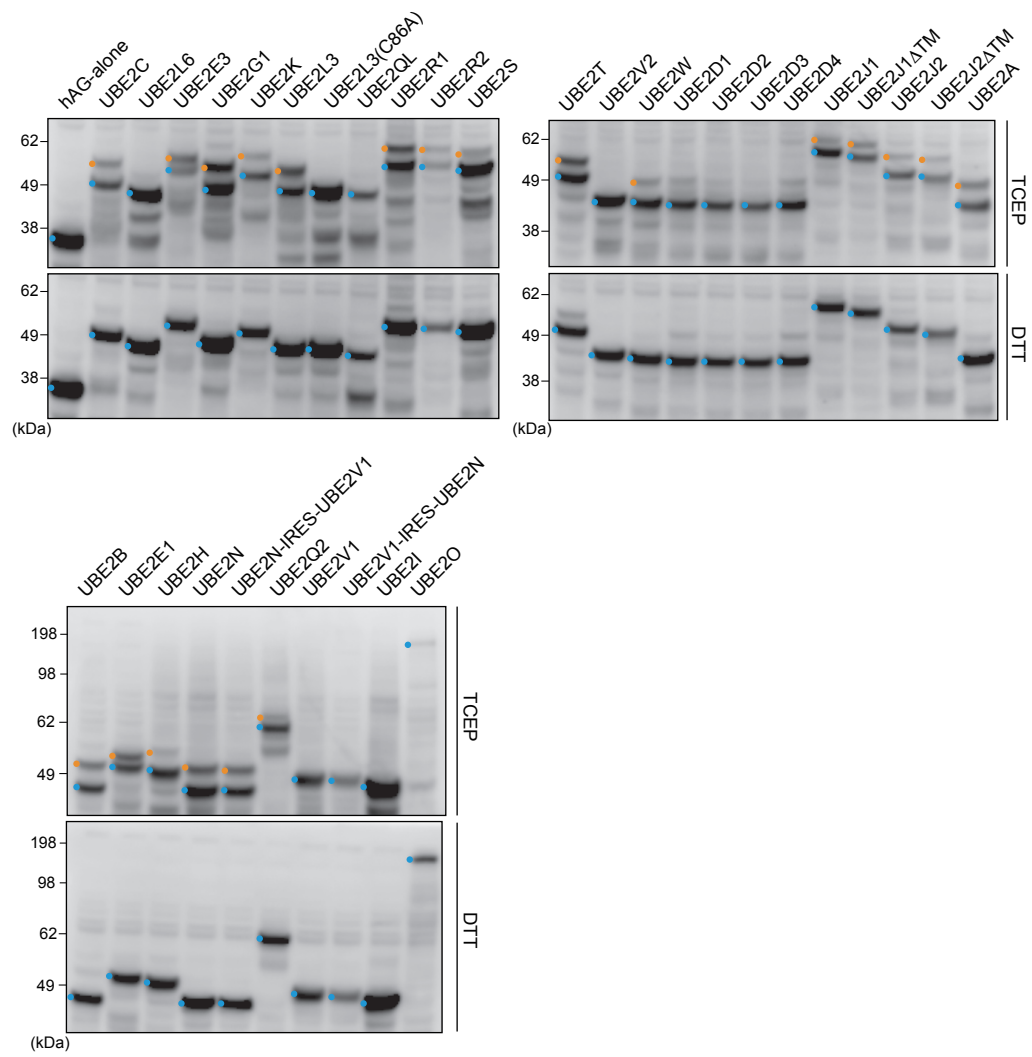

Hayashida et al. Supplemental Figure 1

Supplement: Supplemental Figure S1 — Ubiquitin conjugation of hAG-tagged E2 enzymes. Total cell lysates prepared from HeLa cells expressing the indicated hAG-tagged E2s were electrophoresed in non-reducing (TECP) or reducing (DTT) conditions and then immunoblotted with an anti-Azami Green antibody. Light blue and orange dots denote E2 alone and Ub-conjugated E2, respectively. [file mmc1.pdf]

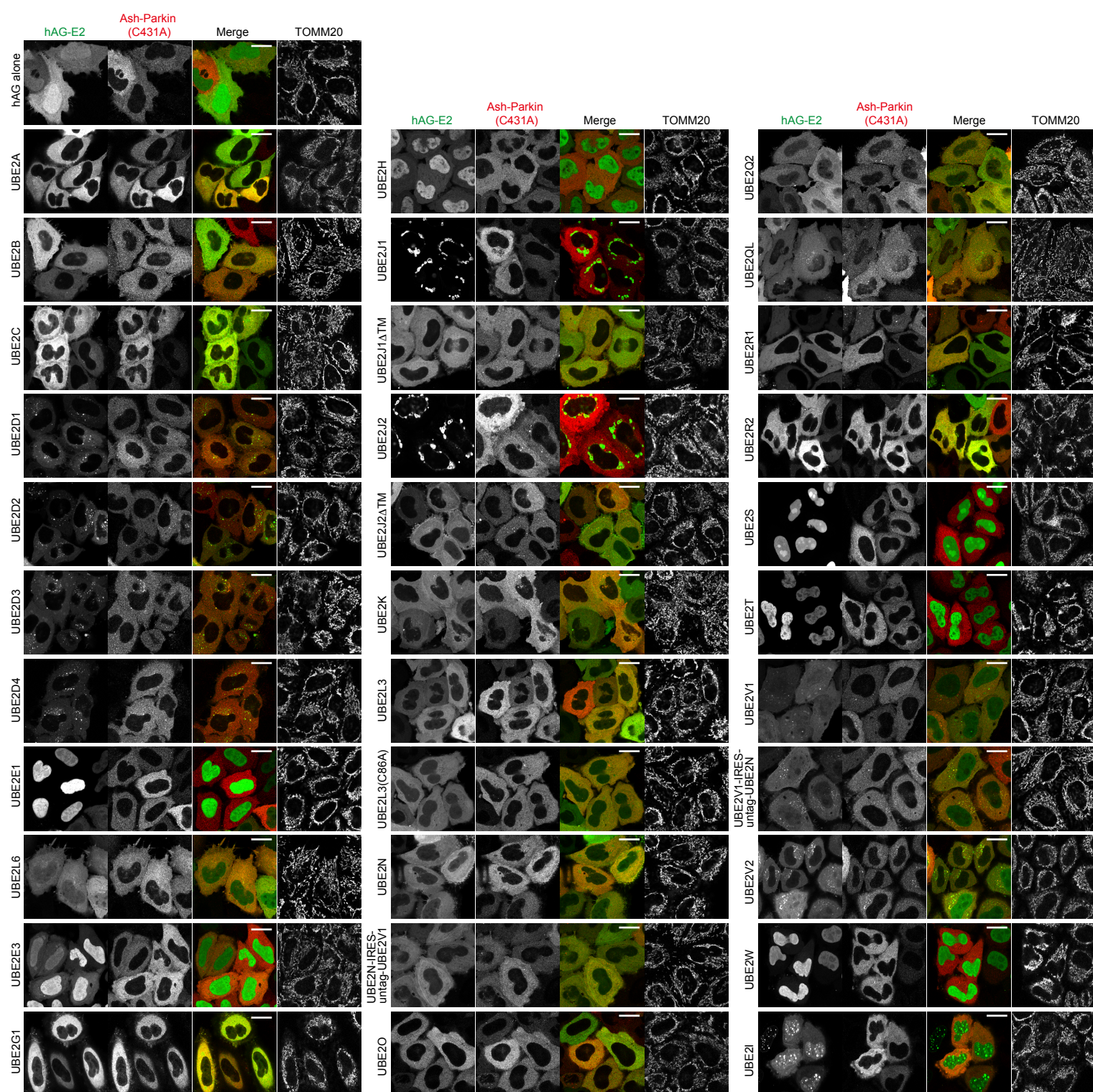

Hayashida et al. Supplemental Figure 2

Supplement: Supplemental Figure S2 — Intracellular localization of hAG-tagged E2s under basal conditions. HeLa cells expressing Ash-Parkin (C431A) and the indicated hAG-tagged E2 enzymes were treated for 3 hrs with DMSO and then immunostained with anti-Parkin and TOMM20 antibodies. Bars, 10 μm. The same merge images are shown in Figure 3B. [file mmc2.pdf]

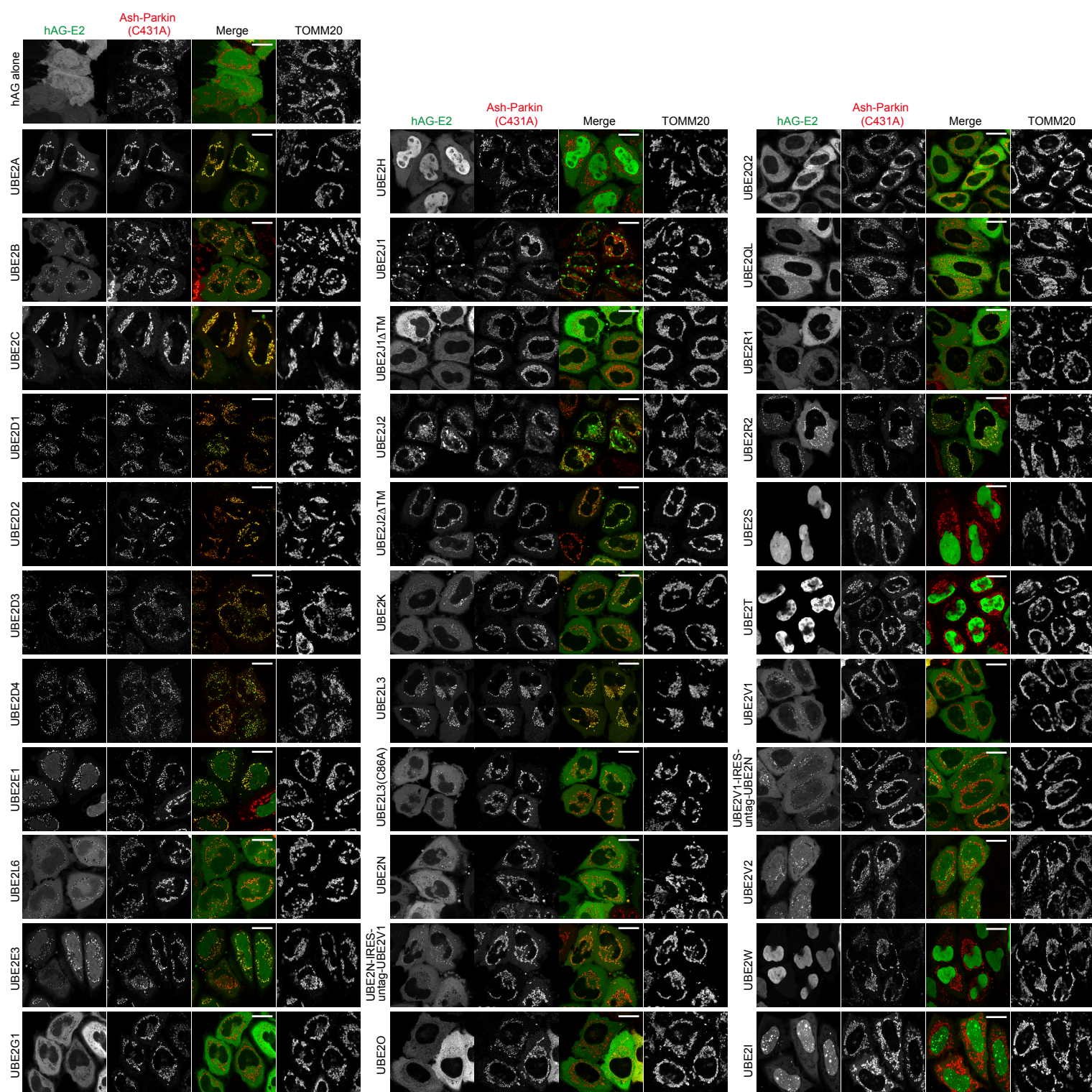

Hayashida et al. Supplemental Figure 3

Supplement: Supplemental Figure S3 — Intracellular localization of hAG-tagged E2s under mitophagy conditions. HeLa cells expressing Ash-Parkin (C431A) and the indicated hAG-tagged E2 enzymes were treated for 3 hrs with valinomycin and then immunostained with anti-Parkin and TOMM20 antibodies. Bars, 10 μm. The same merge images are shown in Figure 3B. [file mmc3.pdf]
